# External iliac artery endofibrosis in an elite female endurance cyclist

**DOI:** 10.1590/1677-5449.200122

**Published:** 2021-04-28

**Authors:** Victor Bilman, Enrico Rinaldi, Francesca Sanvito, Germano Melissano, Roberto Chiesa

**Affiliations:** 1 “Vita-Salute” University, Scientific Institute H. San Raffaele, Division of Vascular Surgery, Milan, Italy.; 2 “Vita-Salute” University, Scientific Institute H. San Raffaele, Division of Anatomic Pathology, Milan, Italy.

**Keywords:** iliac artery, fibrosis, sports medicine, bicycling, artéria ilíaca, fibrose, medicina do esporte, ciclismo

## Abstract

External iliac artery endofibrosis is a rare pathology that affects high-level endurance athletes, especially cyclists. Classical symptoms include pain, loss of power, and/or cramp in the affected limb while training at maximal effort. The patient’s lack of atherosclerotic risk factors makes clinical suspicion of arteriopathy challenging. Moreover, the best management of such patients is still a subject of discussion. We report the case of a 36-year-old professional female endurance cyclist who presented with lower extremity pain during training. Right external iliac artery endofibrosis was diagnosed and the patient underwent surgical treatment. At two-months follow-up, she reported significant improvement in symptoms. This case highlights the importance of diagnosing peripheral vascular disease in young patients and athletes, who do not fit the ordinary profile of patients with atherosclerotic risk factors.

## Resumo

A endofibrose da artéria ilíaca externa é uma doença rara que afeta atletas de resistência (*endurance*) de nível competitivo, principalmente ciclistas. Os sintomas clássicos incluem dor, perda de força e/ou câimbras no membro afetado ao fazer esforço máximo durante o treino. A ausência de fatores de risco ateroscleróticos nesses pacientes torna a suspeita clínica de arteriopatia desafiadora. Além disso, o melhor manejo ainda é motivo de debate. Relatamos o caso de uma ciclista de resistência profissional, de 36 anos, que apresentou dor nos membros inferiores durante o treino. Foi diagnosticada endofibrose da artéria ilíaca externa, e a paciente foi submetida a tratamento cirúrgico. Após seguimento por dois meses, a paciente relatou melhora significativa dos sintomas. Este caso destaca a importância do diagnóstico de doença vascular periférica em pacientes jovens e atletas, os quais não se encaixam no perfil comum do paciente com fatores de risco ateroscleróticos.

## INTRODUCTION

Iliac artery endofibrosis is known as a non-atherosclerotic cause of claudication and/or pain on exertion classically seen in young endurance athletes, especially cyclists.^1^^,^ 2 It was first described in the mid-1980s by Walder et al.^3^ and Chevalier et al.,^4^ who studied bicycle racers with complaints of intermittent claudication in one lower limb during maximum exercise. Its prevalence is still unknown, although, in one prospective study, Schep et al.^5^ estimated that prevalence is as high as 20% amongst professional cyclists.^1^^,^ 6 ^,^ 7 This pathology is often misdiagnosed, which can lead to delayed diagnosis causing anxiety, patient limitations, and unnecessary investigations.^1^

Endofibrosis is characterized by progressive luminal stenosis of the iliac artery, affecting 85% of patients in the external iliac artery (EIA) on only one side.^6^ Despite the fact that, to date, the mechanisms involved in the development of endofibrosis are unknown, several etiological mechanisms have been suspected.^5^^-^ 7 Psoas muscle hypertrophy and the hyperflexed hip joint in the cycling position, in conjunction with long vessel length, leading to repetitive arterial flexion and extension could trigger the development of the endofibrotic reaction in the vessel.^4^^-^ 8 We describe a case of EIA endofibrosis in a world-class female endurance cyclist, who presented with progressive left lower extremity claudication during training, causing professional and social distress.

## CASE REPORT

**Figure 1:**
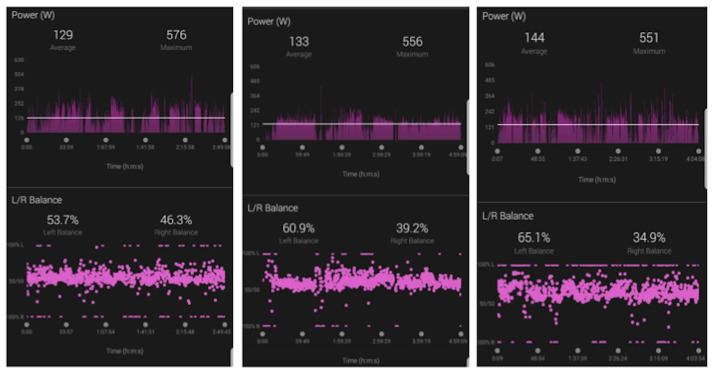
Power balance test: the left-right imbalance became more asymmetrical as the average power balance increased during training. The power output of the left leg is greater than that of the right leg, becoming more evident in more stressful exercises.

A 36-year-old female professional endurance cyclist, with no prior medical history, was referred for right lower extremity claudication during exercise training. She described worsening hip and buttock discomfort at near maximum heart rate, associated with right quadriceps tenderness and weakness. According to her anaerobic threshold test, the point at which lactic acid started to accumulate in her muscles during increasing intensity exercise, i.e. when anaerobic processes become more dominant, was lower in comparison with other endurance athletes. The heart rate corresponding to the ventilatory anaerobic threshold was about 76% of her maximum heart rate (179bpm) (Figure 1). Symptoms were more severe on the right, which made her increase the effort exerted by her left leg during exercises, also leading to pain in both lower limbs. She did not have any symptoms at rest or during moderate efforts. She presented with a dynamic MRI that showed a 5 cm long stenosis of the right EIA, starting at its origin, with bilateral psoas muscle hypertrophy. There was no image of dissection.

Clinical examination was normal, with all pulses palpable bilaterally. Initial at-rest ankle-brachial index (ABI) was normal (ABI right/left, 1.0/1.0). However, a stress ABI showed a significant drop to 0.50 on the right following exercise and a moderate drop to 0.88 on the left, suggesting peripheral artery disease. At rest, Doppler ultrasound (DUS) was normal, but immediately after provocative maneuvers mimicking real-life conditions, flow limitation was confirmed on the side involved. A 2D ultrasound showed homogenous circumferential thickening of the EIA wall.

**Figure 2:**
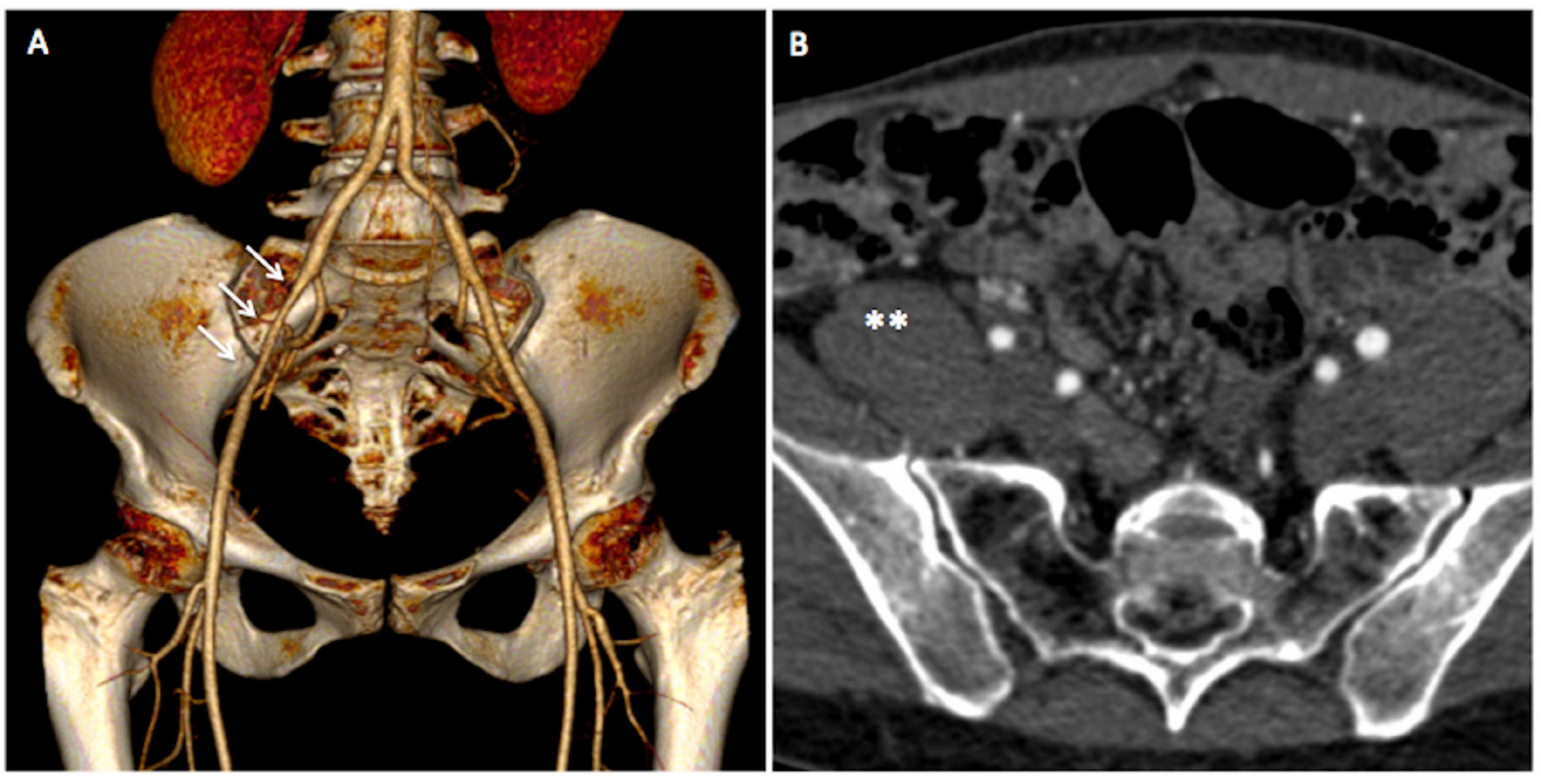
(A) Three-dimensional CT angiographic reconstruction, demonstrating narrowing of the right external iliac artery (arrows); (B) Axial CT image showing right external iliac artery stenosis with a hypertrophic psoas muscle (**).

CT angiography (CTA) showed a regularly patent EIA with uniform reduction of caliber estimated at 40%, with a length of about 5 cm, in the proximal-middle third portion. There was no dissection or post-stenosis dilatation. These findings were compatible with the clinical suspicion of endofibrosis (Figure 2).

**Figure 3:**
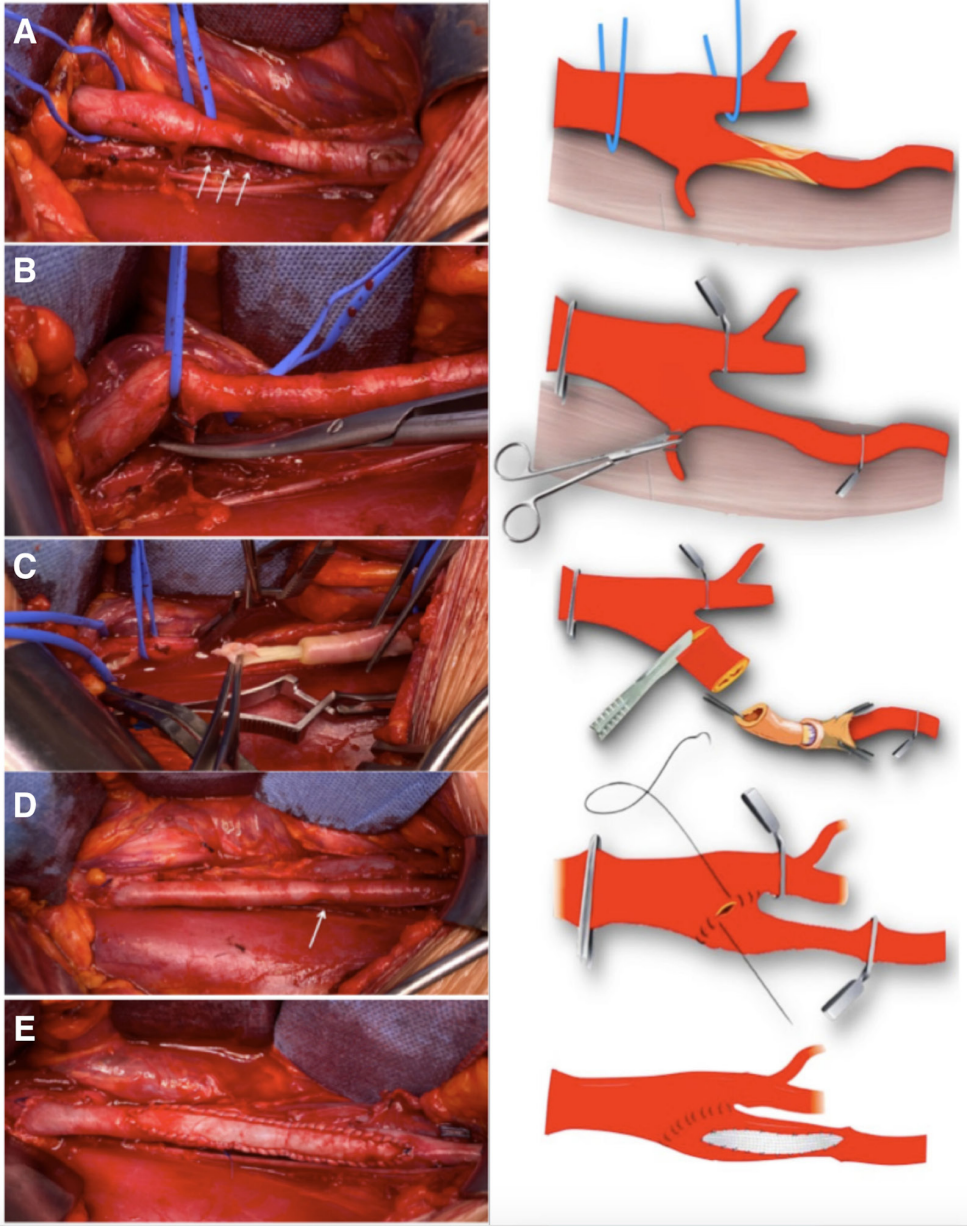
(A) Intraoperative findings of right external iliac artery stenosis distal of its origin (arrows); (B) Releasing the external iliac artery from the muscular arterial branch to the psoas major. This allows the external iliac artery to move freely during exercise and prevents further kinking; (C) Endofibrosis endarterectomy by eversion, known as endofibrosectomy, showing the layer of endofibrosis; (D) External right iliac artery reconstructed with remaining narrowing at end point (arrow); (E) Longitudinal artery incision along its entire length; Enlarged patch angioplasty was performed using a bovine pericardium patch; Final result: good external iliac artery caliber and excellent downstream pulsatility were observed.

Given the patient’s wish to continue competing as a professional cyclist, open surgical management was offered. Surgery was performed through an 8-cm long right pararectal incision with an extraperitoneal approach and no muscle division. The right distal common iliac, as well as the external and internal, iliac arteries were isolated. Stenosis of the EIA of about 6 cm was found. A side branch of the EIA to the psoas muscle was identified, ligated, and divided. After heparinization, the EIA was transected at its origin. Eversion endarterectomy of the EIA was performed and the endofibrosis was revealed and removed. About 2 cm of the EIA was resected, shortening the elongated artery. After re-implanting the EIA by direct end-to-end anastomosis at the bifurcation, a minimal reduction in caliber was still observed in the distal EIA and was confirmed by intraoperative DUS. We, therefore, proceeded with a longitudinal incision along its entire length, and patch angioplasty was performed using bovine pericardium (Synovis Medical, St. Paul, Minnesota). Good EIA caliber and excellent downstream pulsatility were then observed and confirmed with DUS. (Figure 3)

**Figure 4:**
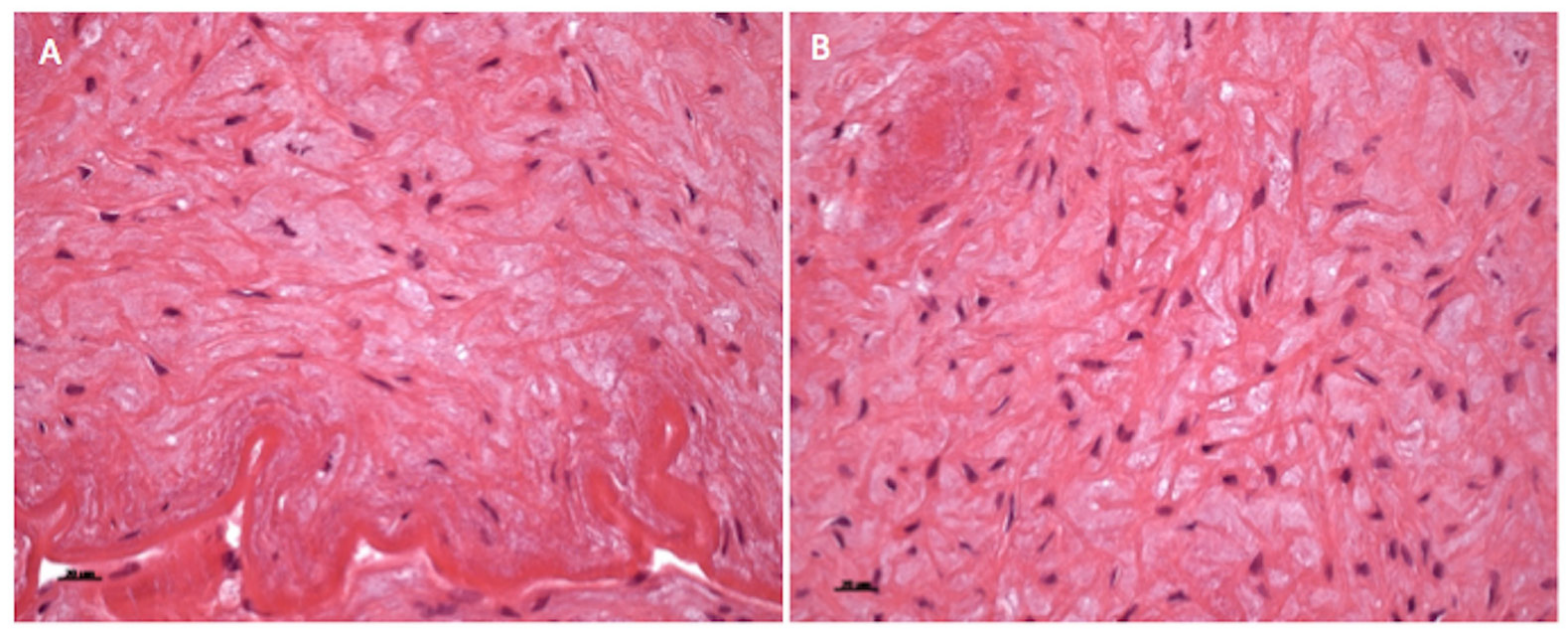
Histopathological findings in the external iliac artery. (A) Hematoxylin and eosin (H&E) staining of endofibrotic lesion showing the loose connective tissue with moderate cellularity of haphazardly arranged stellate or spindle-shaped cells. Arrow indicates the elastic lamina. Scale bar: 50 microns; (B) High magnification of the endofibrotic lesion in A. Scale bar: 20 microns.

Histopathological examination documented the presence of intimal thickening of the EIA, characterized by loose connective tissue endowing haphazardly arranged, stellate, or spindle-shaped cells (Figure 4). Cellularity was moderate to high and a newly formed elastic lamina was observed. These morphological findings confirmed the clinical suspicion of endofibrosis.

The patient had an uneventful recovery and was discharged on postoperative day 3, with long-term aspirin and three months anticoagulation with the novel oral anticoagulant (NOAC) apixaban 2.5mg twice daily. A 2-month postoperative follow-up confirmed excellent clinical conditions. The patient is gradually resuming her usual activities as well as her training.

## DISCUSSION

Iliac artery endofibrosis is an unusual peripheral vascular obstructive disorder that occurs mostly in high-level endurance athletes.^1^^,^ 2 Endofibrosis is characterized by a pathological process of intimal thickening, causing progressive stenosis of the lumen and impaired flow.^6^ An overlying thrombus, dissection, or atherosclerotic infiltration may also be present.^7^ Because this condition is mostly seen in cyclists, some authors suggest that hyperflexion at the hip joint causes repetitive deformation of the EIA such as elongation, torsion, and stretching.^6^^,^ 9 Also, these athletes are known for hypertrophy of the psoas muscle and this may exacerbate the trauma to the vessel by compression and displacement.^6^^,^ 9 Some collateral vessels that anchor the EIAs to the psoas muscle may shorten the mobile portion of the artery and, together with the excessive length of the vessel, constitutes a risk factor for kinking and increased mechanical stress.^10^ Besides all these extrinsic mechanical and anatomical mechanisms, the pathophysiologic mechanism could also be related to intrinsic factors, such as high cardiac output causing shear stress and stimulating endothelial dysfunction.^6^^,^ 9

The histological findings of endofibrosis are quite distinct from those of atherosclerosis, which reveals loose connective tissue with moderate to high cellularity.^6^^,^ 11 Calcification and inflammation are much more present in atherosclerotic lesions, with a major presence of lymphocytes and macrophages. Loose collagen fibers are observed in endofibrosis specimens, whereas the extracellular matrix in atherosclerotic lesions consists of densely packed collagen.^11^ Posthuma et al.^6^ identified collagen and elastin in the neointimal and media layers and postulated a prominent role for myofibroblast-dependent fibrosis. Smooth muscle cell proliferation or differentiation into myofibroblasts increases the alpha-smooth muscle actin, which was demonstrated to have a strong correlation with intraluminal stenosis.^6^ These histological findings distinguish endofibrosis from fibromuscular dysplasia (FMD), which predominantly affects the medial or adventitial layers, and from cystic adventitial disease (CAD), in which mucoid cysts develop in the adventitial layer. Additionally, FMD typically involves renal and extracranial cerebrovascular arteries and CAD mainly involves the popliteal artery.^7^

Endofibrosis is predominantly described in males less than 40 years old, but the greater number of men doing endurance sports may bias this strong association.^7^ Even though arterial endofibrosis is more common in competitive cyclists, it has also been described in other endurance sports such as running, race walking, rugby, and soccer.^7^^,^ 10 The main segment involved is the EIA, in 90% of the cases, with the endofibrotic segment measuring between 2 to 6 cm. Involvement of common iliac, common femoral, or profunda femoris arteries has also been reported.^7^

When history and examination are not suggestive of musculoskeletal or neurological causes, a vascular cause must be investigated. At rest, the ankle-brachial index (ABI) is usually normal, but it may be possible to identify endofibrotic flow limitation after 5 minutes of maximal exercise.^7^ Although ABI has a sensitivity and specificity of up to 100%, Doppler ultrasound also proves effective for determining the location of the disease.^12^ CTA and MRI enable accurate assessment of the diameter and length of endofibrosis, and digital subtraction angiography is useful for detecting arterial branches to the psoas muscle and for better evaluation of severe stenosis or occlusion.^7^^,^ 10

Management of this pathology involves a variety of modalities.^1^^,^ 7 ^,^ 10 The vascular surgeon will feel conflicted with regard to the best management approach in such young and athletic patients. As emphasized by Hinchliffe,^13^ in athletes, the demands imposed on vascular surgical reconstruction, such as long-term-patency or durability, far outweigh those that may be required or considered normal for most patients within standard clinical vascular practice. Conservative measures should be used before any surgical intervention; however, untreated stenosis may predispose patients to atherosclerotic disease. If training is stopped, there is some evidence that endofibrotic lesions may stabilize.^7^^,^ 10

Endovascular procedures such as angioplasty only or stenting have been associated with high rates of recurrence, due to recoil, intimal hyperplasia, or dissection of the endofibrotic segment.^7^^,^ 14 Stent fracture, migration, and plicature have also been described.^9^ Although good short-term outcomes have been described with endovascular repair, symptom recurrence occurred in a significant number of patients.^7^ Unsatisfactory long-term endovascular outcomes could be explained by the fact that the mechanical forces inherent to the condition are still acting, resulting in continuous arterial damage.

Surgical repair is a definitive treatment for this condition.^7^ In addition to the fact that the surgical treatment removes the endofibrotic segment and restores the lumen caliber to normal, the release of arterial branches to the psoas muscle and shortening of the EIA prevent further arterial kinking. Schep et al.^5^ demonstrated that release of restrictive fibrous tissue and any arterial branch of the EIA to the psoas could in isolation be beneficial to patients.^,^ 7 While endofibrosis excision and primary anastomosis are the preferable management, iliofemoral bypass grafts are an acceptable and effective alternative for patients with complex lesions, achieving higher long-term patency rates than endovascular repair.^8^

Although it has already been demonstrated that open repair can allow high-level athletes to return to elite sport, there is not enough information to establish long-term effectiveness. Therefore, advantages and drawbacks should be thoroughly discussed with the patient.^13^

## CONCLUSION

Iliac artery endofibrosis can have a considerable impact on the lives of high-level athletes, who desire relief from symptoms. In endurance athletes with symptoms of claudication, a high index of suspicion of endofibrosis should be maintained. Conservative therapies such as physiotherapy appear to be ineffective, as do endovascular procedures. To date, surgical repair is the only definitive treatment, but long-term outcomes still need to be evaluated.
